# Pharmacodynamics and Medicinal Chemistry of an External Chinese Herbal Formula for Mammary Precancerous Lesions

**DOI:** 10.1155/2017/6235027

**Published:** 2017-07-24

**Authors:** Ruixue Chen, Guijuan Zhang, Yi Ma, Fengjie Bie, Hongxia Fan, Min Ma

**Affiliations:** ^1^School of Chinese Medicine, Jinan University, Guangzhou, Guangdong 510632, China; ^2^The First Affiliated Hospital of Jinan University, Guangzhou, Guangdong 510630, China; ^3^Institute of Biomedicine, Department of Cellular Biology, Guangdong Province Key Lab of Bioengineering Medicine, Jinan University, Guangzhou, Guangdong 510632, China; ^4^College of Pharmacy, Jinan University, Guangzhou, Guangdong 510632, China

## Abstract

Ruyan Neixiao Cream (RYNXC) is a traditional Chinese herbal formula for treating mammary precancerous disease. This study was carried out to investigate in vivo anticancer effect of RYNXC and multiple constituents. 32 virginal Sprague-Dawley rats were randomly divided into blank control group (BC), mammary precancer models group (MODEL), tamoxifen group (TAM), and Ruyan Neixiao Cream group (RYNXC). TAM was intervened by tamoxifen; RYNXC was intervened by Ruyan Neixiao Cream. The chromatographic separation was performed by high performance liquid chromatography (HPLC) coupled with mass spectrometry (MS). RYNXC showed significant improvement in erythrocyte aggregation index (EAI), hematocrit (HCT), fibrinogen (FIB), spleen coefficient, and uterus coefficient compared with MODEL. In RYNXC and TAM groups, atypical hyperplasia was observed in pathological mammary tissues; meanwhile in MODEL group, ductal carcinoma was observed in situ. Moreover, fifteen compounds were characterized according to HPLC-MS data, including organic acids, tannin, alkaloid, volatile oil, anthraquinones, and flavonoids. The study suggests that RYNXC was an effective Chinese herbal formula for mammary precancerous lesions and provides a scientific basis for the quality standard and the pharmacology of RYNXC. It will be beneficial to the future clinical application of RYNXC.

## 1. Introduction

Breast cancer has been ranked first in women's malignancy around the world, while the mortality and morbidity were increasing [1]. The American Society of Clinical Oncology (ASCO) recommended aromatase inhibitor (AI) for 5 years or AI for 2 to 3 years and changing into tamoxifen (TAM) for 5 years in all as initial choice to patients who were diagnosed with hormone-receptor-positive breast cancer. A clinical study from University of Oxford reported that, for T1N0 patients with low, middle, and high grade, the risks of distant recurrence in 5~14 years were 5%, 8%, and 10%, and the local recurrence rates were 12%, 15%, and 17%; even with endocrine therapy for 5 years, the recurrence risks remained [2]. World Health Organization (WHO) reported that precancerous lesions were diseases in which the risks developing into cancer were more than 20%. It is known that the development of breast cancer is of multiple stages: benign hyperplasia, atypical hyperplasia, followed by carcinoma in situ, and finally invasive carcinoma [3, 4]. That is to say, diseases may develop into cancer at the stages of atypical hyperplasia and carcinoma in situ [5].

In this study, rats' model of mammary precancerous lesions was established by DMBA plus estrogen and progesterone. DMBA is a commonly used chemical carcinogen for chemical induction of mammary cancer model [6]. Breast is a target organ of hormone; the incidence of breast cancer may be increasing with the increasing levels of estrogen and progesterone; therefore, rats' model of mammary precancerous lesions was established by regulating intervention time and dosage of estrogen and progesterone [7].

Studies showed that therapies could effectively reduce the incidence of malignant tumor, for example, traditional Chinese medicine (TCM) [8, 9]. In TCM, breast precancerous lesion is considered equivalent to the term “Ru Pi,” which was described in the “General Treatise on the Cause and Symptoms of Diseases” (Chinese name in pinyin is “Zhu Bing Yuan Hou Lun”) in the Sui Dynasty. TCM is characterized by syndrome differentiation. In this regard, “depression of liver and deficiency of spleen, stasis of blood, and thoroughfare-controlling disharmony” are considered to be the basic pathogenic factors of mammary precancerous lesions. Therefore, relieving the suppressed liver and replenishing the spleen energy, activating blood circulation, and regulating the thoroughfare for anticancer treatment are the most important therapies in the treatment of mammary precancerous lesions.

Ruyan Neixiao Cream (RYNXC) was invented by School of Chinese Medicine (patent applied number: 201110029344.1), Jinan University (Guangzhou, China). The herbs in RYNXC were modified from the well-known traditional Chinese prescription “Yindu Neixiao Pulvis” which originated from the “Yao Lian Qi Mi” in 1920–1940 and is a Chinese herbal formula that has been used for a long time in the external treatment of surgical diseases, including breast cancer. RYNXC consists of multiple traditional Chinese herbs that have been widely used in oral decoction in clinic. However, the external preparation of RYNXC has not been applied. Therefore, pharmacological effect and chemical constituents of RYNXC are required so as to popularize its clinical application.

## 2. Materials and Methods

### 2.1. Reagents and Preparation

7,12-Dimethylbenz[a]anthracene (DMBA, Tokyo Chemical Industry Co., Ltd., Japan) was dissolved in sesame oil by 7 mg/ml ratio, followed by Estradiol Benzoate injection, 1 ml/2 mg (Shanghai GM Pharmaceutical Co., Ltd., China), and progesterone injection, 1 ml/10 mg (Zhejiang Xianju Pharmaceutical Co., Ltd., China). Tamoxifen was purchased from Chi-Fei Chemical Co., Ltd. (Wuhan, China). Ointment was prepared by PEP4000, PEP400, and propylene glycol. Methanol was of analytical reagent grade (Sigma-Aldrich, St. Louis., MO, USA). Formic acid was of HPLC grade (Dikma Technologies, USA). HPLC-grade water was prepared using a Milli-Q water purification system (Millipore, USA).

**Figure 1:**
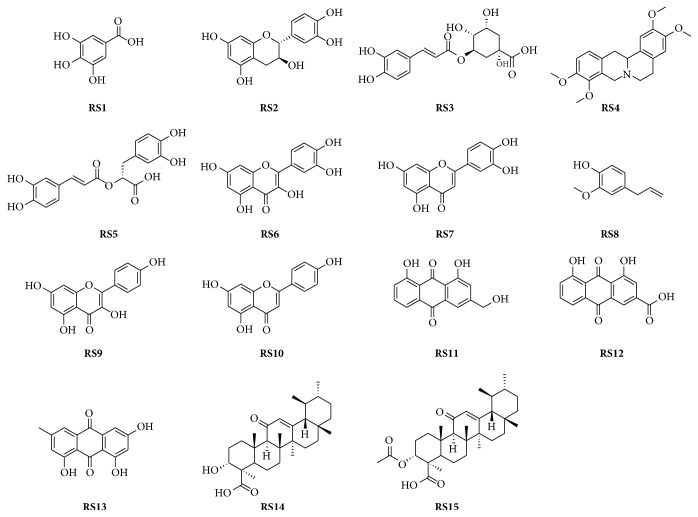
Chemical structures of the reference substances (RS).

The herb extractum of RYNXC was prepared in School of Chinese Medicine, Jinan University (Guangzhou, China). Fifteen standards were used as reference substances (RS): gallic acid (RS1), cianidanol (RS2), chlorogenic acid (RS3), tetrahydropalmatine (RS4), rosmarinic acid (RS5), quercetin (RS6), luteolin (RS7), eugenol (RS8), kaempferol (RS9), apigenin (RS10), aloe-emodin (RS11), rhein (RS12), emodin (RS13) (Chengdu Pufei De Biotech Co., Ltd., China), 11-keto-*β*-boswellic acid (RS14) (Beijing Zhongke Yiyou Institute of Chemical Technology, China), and 3-acetyl-11-keto-*β*-boswellic acid (RS15) (Shanghai Yuanye Biotechnology Co., Ltd., China). The purity of all the reference substances is ≥98% and the chemical structures are shown (Figure 1). Stock solutions (200 *μ*g/ml) of the RS were prepared by dissolving in methanol and storing at 4°C until use.

Powdered samples of all herbs were prepared. Firstly, all the herbs' powders were mixed and heated for reflux extraction by 60% ethanol water with the ratio of 1 : 10 (w : v) for 90 minutes; then the residue was filtered and decocted with 60% ethanol water twice as the first time. The filtrate was removed by a rotary evaporation apparatus and then dried in a vacuum oven to form the dry extractum. The dried extractum was extracted with ether with the ratio of 1 : 30 (w : v) in an ultrasonic water bath for 10 min at 37°C with 1 hour of standing twice. The ether extracts were combined and evaporated until dryness. Then the residue was dissolved in MeOH with the ratio of 1 : 10 (w : v). At last, the solution was centrifuged at 12000 r/min for 5 minutes and filtered through a Strata™ C18-E solid-phase extraction (SPE) column (55 *μ*m, 70 A, Phenomenex, USA) before injection into the HPLC system.

### 2.2. Instrumentation

Instrumentation included biological microscope (Leica DM6000B, Germany), slicing machine (Leica AS-325, Germany), slide drier (Leica 202-2, Germany), centrifugal machine (Sigma 2-16P, Beijing, China), Milli-Q Academic System (Millipore, USA), Dionex UltiMate 3000 Rapid Separation LC system (Dionex, USA), Thermo Scientific Dionex UltiMate 3000 Rapid Separation Diode Array Detector, an evaporative light scattering detector (Alltech, USA), and AmaZon SL electrospray ionization ion trap mass spectrometer (Bruker Daltonics, GER).

### 2.3. Animals

32 SPF level 8-week-old virginal Sprague-Dawley rats (weight: 200–250 g) were purchased from Experimental Animal Center, Guangzhou University of Traditional Chinese Medicine (approval number SCXK (Yue) 2013-2234). All the animals were maintained in an environmentally controlled clean air room with 12-hour light and 12-hour dark cycle, with temperature of 20~25°C and relative humidity of 60–90%. The rats were fed whole value grain feedstuff and were given access to tap water ad libitum. Treatment began one week after arrival. The animal experiments were conducted in accordance with the Guide for the Care and Use of Laboratory Animals (2008, Washington, DC). The protocols for the animal studies were also reviewed and approved by the Experimental Animal Ethics Committee of Jinan University.

The rats were randomly divided into blank control group (BC, *n* = 8), mammary precancer models group (MODEL, *n* = 8), tamoxifen group (TAM, *n* = 8), and Ruyan Neixiao Cream group (RYNXC, *n* = 8). Before cyclic drugs, BC group was given a single gavage of sesame oil (1 mg/100 g); MODEL, TAM, and RYNXC groups were given the same dosage of sesame oil with DMBA 7 mg. Then, for 5 days per cycle, BC and MODEL groups were smeared ointment (0.2 g) with nothing around the breasts, TAM group was smeared ointment with tamoxifen (0.2 g), and RYNXC group was smeared ointment with Ruyan Neixiao Cream (0.2 g) each day. Meanwhile, at first 3 days, BC group was injected with oil (0.25 ml/kg) and MODEL, TAM, and RYNXC groups were injected with Estradiol Benzoate (0.5 mg/kg). On the 4th day, BC group was injected with oil (0.4 ml/kg) and MODEL, TAM, and RYNXC groups were injected with progesterone (4 mg/kg). On the 5th day, there was only observation.

### 2.4. Detection of Indexes

All rats' physiological conditions, symptoms, and signs of drug toxicity were daily observed, including appetite, hair, weight, and nipple color and shape. After 60 days' intervention, the microcirculation perfusion of rats' breast tissues was observed. Blood was obtained from the abdominal aorta for the observation of hemorheology. The spleen tissues, liver tissues, and uterus tissues were rapidly removed and washed with saline at 4°C. Breast tissues were dissected and fixed for 24 hours in 10% neutral buffered formalin and then stained with hematoxylin-eosin (H&E) staining and classified according to criteria of references. The remaining tissues were stored at −80°C for later use.

HPLC was performed on a COSMOSIL 5C18-MS-II reverse-phase column (4.6 mm × 250 mm, 5 *μ*m, Nacalai, Japan). The mobile phase consisted of 0.1% (v/v) formic acid in water (A) and methanol (B). A gradient programme was used as follows: 0–46 min, 35–53% B; 46–56 min, 53%–68% B; 56–71 min, 68–80% B; 71–81 min, 80–90% B; 81–91 min, 90–100% B; and 91–111 min, 100% B. A 15-minute postrun equilibration back to the initial mobile phase composition was included after each analysis. All mass spectra were acquired in the positive and negative ion modes. The capillary was at 4500 V, the end plate offset was 500 V, the nebulizer was 15.0 psi, dry gas was provided at 8.0 L/min, the dry gas temperature was 200°C, and the scan range was 50–2200 *m*/*z*.

### 2.5. Statistical Analysis

All data were analyzed by SPSS 17.0 and Microsoft Excel 2007. Experimental data were analyzed by One-Way ANOVA Test and the experimental data were expressed as mean and standard deviation ($\overset{-}{X}\pm SD$). Homogeneity of variances was conducted by two-tailed test. If the variances were homogeneous (*P* > 0.1), Newman-Keuls test was used to calculate the experimental data. If the variances were not homogeneous (*P* < 0.1), Tamhane's T2 test was used to calculate the experimental data. *P* value < 0.05 was considered statistically significant.

## 3. Results

### 3.1. Pharmacodynamics of RYNXC

#### 3.1.1. Microcirculation Perfusion of Mammary Tissues

The nipple numbers of each group were 16. It was shown that the microcirculation perfusion of mammary tissues among groups was significantly different (*F *= 3.84;* P *= 0.0139). MODEL, TAM, and RYNXC groups showed significant decrease compared with BC group (*P* < 0.05). There was no significant difference among MODEL, TAM, and RYNXC groups (*P* > 0.05) (Table 1).

#### 3.1.2. Hemorheology

It was shown that the hematocrit (HCT) among groups was significantly different (*F *= 107.49;* P *= 0.000). MODEL, TAM, and RYNXC groups showed significant increase compared with BC group (*P *= 0.000). TAM and RYNXC groups showed significant decrease compared with MODEL group (*P* < 0.05). RYNXC group showed significant decrease compared with TAM group (*P* = 0.024).

It was shown that the fibrinogen (FIB) among groups was significantly different (*F *= 33.05;* P *= 0.000). MODEL, TAM, and RYNXC groups showed significant increase compared with BC group (*P* < 0.05). TAM and RYNXC groups showed significant decrease compared with MODEL group (*P* = 0.000). There was no significant difference between TAM and RYNXC groups (*P* = 0.126).

It was shown that the erythrocyte aggregation index (EAI) among groups was significantly different (*F *= 7.34;* P *= 0.001). MODEL group showed significant increase compared with BC group (*P* = 0.001). TAM and RYNXC groups showed no significant increase compared with BC group (*P* > 0.05). TAM and RYNXC groups showed significant decrease compared with MODEL group (*P* = 0.000). There was no significant difference between RYNXC and TAM groups (*P* = 0.093) (Table 2).

#### 3.1.3. Organ Coefficient

It was shown that the spleen coefficient among groups was significantly different (*F *= 7.40;* P *= 0.001). MODEL group showed significant increase compared with BC group (*P* = 0.012). TAM and RYNXC groups showed no significant difference compared with BC group (*P* > 0.05). TAM and RYNXC groups showed significant decrease compared with MODEL group (*P* < 0.05). There was significant difference between TAM and RYNXC groups (*P* = 0.048).

It was shown that the liver coefficient among groups was not significantly different (*F *= 2.07;* P *= 0.1264). MODEL, TAM, and RYNXC groups showed no significant increase compared with BC group (*P* > 0.05). TAM and RYNXC groups showed no significant difference compared with MODEL group (*P* > 0.05). There was no significant difference between TAM and RYNXC groups (*P* = 0.076).

It was shown that the uterus coefficient among groups was significantly different (*F *= 149.01;* P *= 0.000). Compared with BC group, MODEL group showed significant increase (*P* = 0.000), TAM group showed significant decrease (*P* = 0.002), and RYNXC group showed no significant difference (*P* = 0.890). TAM and RYNXC groups showed significant decrease compared with MODEL group (*P* = 0.000). And there was significant difference between TAM and RYNXC groups (*P* = 0.010) (Table 3).

#### 3.1.4. Pathological Observation

**Figure 2:**
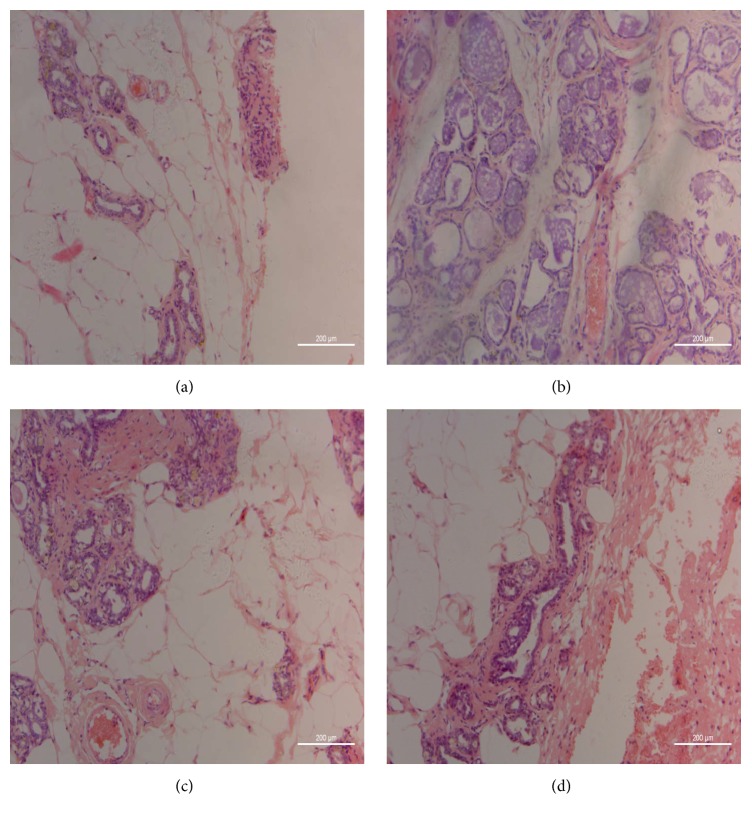
Mammary tissues (HE ×200). (a) BC. (b) MODEL. (c) TAM. (d) RYNXC.

H&E staining was performed. Invasive carcinoma was not found in pathological observations of all rats' mammary tissues. Ductal carcinoma was observed in situ in MODEL group, and atypical hyperplasia was observed in TAM and RYNXC groups (Figure 2).

### 3.2. Medicinal Chemistry of RYNXC

#### 3.2.1. Optimization of the HPLC Conditions

**Figure 3:**
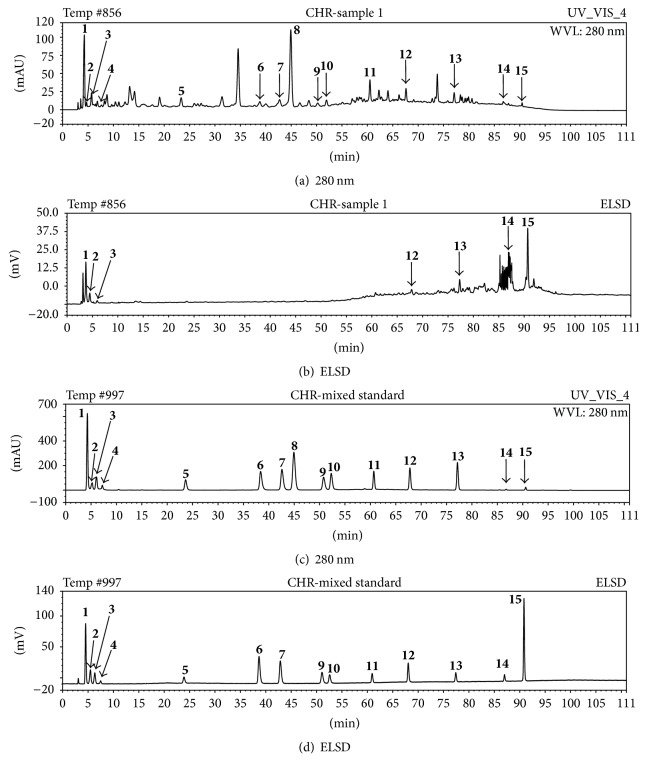
(a) HPLC chromatogram of the extract in RYNXC at 280 nm. (b) HPLC chromatogram of the extract in RYNXC by ELSD. (c) HPLC chromatogram of fifteen reference substances at 280 nm. (d) HPLC chromatogram of fifteen reference substances by ELSD.

The extract of RYNXC was injected into HPLC system. Sample volume was 10 *μ*L, flow rate was 0.8 mL/min, and column temperature was 30°C. Five wavelengths (208, 220, 254, 280, and 365 nm) were tested for the best detection sensitivity, although all the compounds have different UV absorption characteristics, and the detection wavelength of 280 nm gave the optimal results except for peaks 14 and 15, in which evaporative light scattering detector (ELSD) gave more accurate detection. Moreover, the ELSD chromatogram of the extract in RYNXC showed that several compounds that had low response were not marked (compounds 4–11) (Figure 3).

#### 3.2.2. Characterization of the Compounds in RYNXC

**Figure 4:**
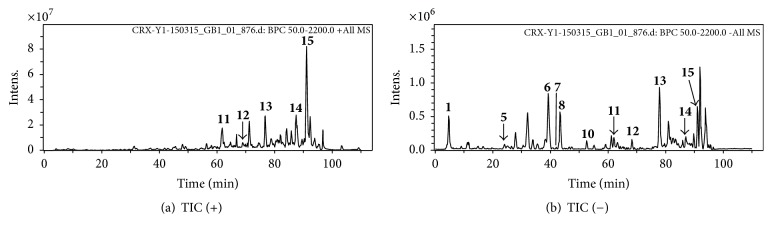
Total ion chromatogram of the extract in RYNXC. (a) Positive mode. (b) Negative mode.

Peaks are separated and detected according to the spectrum of each peak in the total ion chromatograms (TICs). In the TICs, some ion peaks of compounds which had low intensity were not marked in the figure (compounds 2–4 and 9) (Figure 4). Among them, fifteen compounds, 5 organic acids, 1 tannin, 1 alkaloid, 1 volatile oil, 3 anthraquinones, and 4 flavonoids, were tentatively identified. Fifteen compounds were detected by DAD and ELSD. The compounds were all reported previously from the ingredient herbs of RYNXC and were tentatively characterized by comparison with reference substances based on their HPLC retention time and MS fragmentation patterns. The features of the ESI-MS data were mainly [M−H]^−^ ions, [M+H]^+^ ions, and [M+Na]^+^ ions. The retention times, molecular weights, identification, and formulas are presented in Table 4.

## 4. Discussion

In this pharmacodynamics study, RYNXC group showed significant improvement in HCT (*P* < 0.05), FIB (*P* < 0.05), EAI (*P* < 0.05), spleen coefficient (*P* < 0.05), and uterus coefficient (*P* < 0.05) compared with MODEL group. And in pathological observation, ductal carcinoma was observed in situ in MODEL group and atypical hyperplasia was observed in RYNXC and TAM groups. Therefore, RYNXC was an effective Chinese herbal formula for mammary precancerous lesions.

The compatibility of traditional Chinese medicine refers to the combination of multiple herbs, based on clinical effects and the properties of herbs. The formulating herbs in RYNXC have effects of relieving the suppressed liver, activating blood circulation, and regulating the thoroughfare. It is important to study medicinal chemistry of RYNXC so as to understand its biological and medical significance. However, chemical analysis and quality control studies on RYNXC are limited. HPLC-MS is popular for direct identification of multiple components and for the quality control of Chinese herbs because it has wide suitability and sensitivity and provides sufficient structural information. DAD supports peak characterization of the sharpest peaks of fast separations for reliable sample data. ELSD could detect sample concentration with or without UV absorption. ESI can lead to only protonated or deprotonated molecules [10, 11]. Therefore, HPLC-ESI-MS is one of the most efficient analytical techniques available for the analysis of Chinese herbal formulas and provides a rapid method to separate and identify multiple constituents in RYNXC.

Modern phytochemistry studies showed that herbs in RYNXC contain multiple bioactive constituents. This study identified fifteen compounds in RYNXC: gallic acid, cianidanol, chlorogenic acid, tetrahydropalmatine, rosmarinic acid, quercetin, luteolin, eugenol, kaempferol, apigenin, aloe-emodin, rhein, emodin, 11-keto-*β*-boswellic acid, and 3-acetyl-11-keto-*β*-boswellic acid. The compounds can be classified as organic acids, tannin, alkaloid, volatile oil, anthraquinones, and flavonoids. Among them, volatile oils have been commonly accepted as bioactive constituents in pharmacology, such as eugenol, which was confirmed to have tumor suppression effect [12, 13].

Anthraquinone, tannins, and polysaccharide were confirmed to inhibit proliferation of cancer cell and induce apoptosis [14]. Gallic acid and cianidanol were confirmed to have antitumor and antioxidant effects [15, 16]. Alkaloids, such as tetrahydropalmatine, have been approved to inhibit proliferation of cancer cells [17]. Flavonoids are approved as bioactive components, such as apigenin, which was confirmed to inhibit cancer cell proliferation, migration, and invasion [18]. Flavonoid glycosides were confirmed to have antioxidant effect [19]. Boswellic acids, including 11-keto-*β*-boswellic acid (KBA) and 3-acetyl-11-keto-*β*-boswellic acid (AKBA), were approved to have the effect of inhibiting proliferation of cancer cell [20, 21]. Eugenol in* Commiphora myrrha* Engl. is a typical active constituent. The extract of* Commiphora myrrha* Engl. showed significant cytotoxicity of breast cancer cells [22, 23].

In summary, the study revealed that RYNXC was efficacious in improving HCT, FIB, EAI, spleen coefficient, and uterus coefficient and inhibiting the development of carcinoma in mammary tissues. Therefore, RYNXC is an effective Chinese herbal formula for mammary precancerous lesions. Moreover, we identified fifteen compounds in RYNXC: gallic acid, cianidanol, chlorogenic acid, tetrahydropalmatine, rosmarinic acid, quercetin, luteolin, eugenol, kaempferol, apigenin, aloe-emodin, rhein, emodin, 11-keto-*β*-boswellic acid, and 3-acetyl-11-keto-*β*-boswellic acid. This study provides a scientific basis for the quality standard and pharmacodynamics of RYNXC. It will be helpful to the clinical application of RYNXC for treating mammary precancerous lesions in the future.

## Figures and Tables

**Table 1:** Results of microcirculation perfusion (*n* = 16, $\overset{-}{X}\pm SD$).

Group	Microcirculation perfusion
BC	163.94 ± 44.57
MODEL	121.22 ± 28.01^△^
TAM	128.48 ± 50.04^△^
RYNXC	131.2 ± 26.64^△^

^△^ *P* < 0.05 versus BC.

**Table 2:** Results of hemorheology in rats (*n* = 8, $\overset{-}{X}\pm SD$).

Group	HCT	FIB	EAI
BC	0.3 ± 0.02	2.03 ± 0.09	2.36 ± 0.11
MODEL	0.46 ± 0.02^△^	2.88 ± 0.21^△^	2.51 ± 0.03^△^
TAM	0.40 ± 0.02^△▲^	2.21 ± 0.17^△▲^	2.42 ± 0.05^▲^
RYNXC	0.38 ± 0.01^△▲^	2.37 ± 0.22^△▲^	2.36 ± 0.08^▲^

^△^ *P* < 0.05 versus BC. ^▲^*P* < 0.05 versus MODEL.

**Table 3:** Results of organ coefficients in rats (*n* = 8, $\overset{-}{X}\pm SD$).

Group	Spleen	Liver	Uterus
BC	1.79 ± 0.47	27.80 ± 1.75	2.89 ± 0.87
MODEL	3.08 ± 1.18^△^	31.59 ± 5.22	20.53 ± 3.84^△^
TAM	1.65 ± 0.27^▲^	31.21 ± 4.69	1.44 ± 0.58^△▲^
RYNXC	2.00 ± 0.37^▲^	28.49 ± 1.95	2.87 ± 1.36^▲^

^△^ *P* < 0.05 versus BC. ^▲^*P* < 0.05 versus MODEL.

**Table 4:** Identification of the multiple constituents in RYNXC.

Analyte	*T*_*R*_ (min)	Parent ions *m*/*z*	M.W. (Da)	ID	Formula
**1**	4.27	[M−H]^−^ 168.9, [M+H]^+^ 170.9	170.12	Gallic acid	C_7_H_6_O_5_
**2**	5.32	[M−H]^−^ 289.0, [M+H]^+^ 291.0	290.27	Cianidanol	C_15_H_14_O_6_
**3**	6.02	[M−H]^−^ 353.1, [M+H]^+^ 355.1	354.31	Chlorogenic acid	C_16_H_18_O_9_
**4**	7.89	[M+H]^+^ 356.1	355.43	Tetrahydropalmatine	C_21_H_25_NO_4_
**5**	23.52	[M−H]^−^ 359.5, [M+Na]^+^ 383.2	360.31	Rosmarinic acid	C_18_H_16_O_8_
**6**	38.14	[M−H]^−^ 301.3, [M+H]^+^ 303.3	302.24	Quercetin	C_15_H_10_O_7_
**7**	42.44	[M−H]^−^ 285.0, [M+H]^+^ 287.0	286.24	Luteolin	C_15_H_10_O_6_
**8**	45.22	[M**+**102]^+^ 266.0	164.2	Eugenol	C_10_H_12_O_2_
**9**	50.68	[M−H]^−^ 285.4, [M+H]^+^ 287.1	286.24	Kaempferol	C_15_H_10_O_6_
**10**	52.44	[M−H]^−^ 268.9, [M+H]^+^ 270.9	270.24	Apigenin	C_15_H_10_O_5_
**11**	60.70	[M−H]^−^ 269.6, [M+H]^+^ 270.9	270.24	Aloe-emodin	C_15_H_10_O_5_
**12**	67.75	[M−H]^−^ 283.1, [M+H]^+^ 284.9	284.22	Rhein	C_15_H_8_O_6_
**13**	77.18	[M−H]^−^ 269.0, [M+H]^+^ 271.0	270.24	Emodin	C_15_H_10_O_5_
**14**	86.61	[M−H]^−^ 470.7, [M+H]^+^ 471.3	470.68	KBA	C_30_H_46_O_4_
**15**	90.32	[M+H]^+^ 513.3, [2M−H]^−^ 1024.6	512.72	AKBA	C_32_H_48_O_5_
